# A Rare Case of Myoepithelioma around the Left Orbit

**DOI:** 10.5681/joddd.2011.032

**Published:** 2011-12-19

**Authors:** Kapila Rishabh, Trivedi Ashwarya, Sudhir R

**Affiliations:** ^1^Senior Lecturer, Department of Oral Medicine and Radiology, Guru Nanak Dev Dental College and Research Institute, Sunam, Punjab, India; ^2^Lecturer, Department of Pediatric Dentistry, D.A.P.M.R.V. Dental College and Hospital, Bangalore, Karnataka, India

**Keywords:** Myoepithelioma, orbit, neoplasm, salivary

## Abstract

Myoepithelioma is a rare neoplasm of the salivary glands, generally occurring in the parotid gland and less often in the mi-nor accessory salivary glands of the oral cavity. It is known to be a rare entity occurring at unusual locations, which makes it difficult to diagnose. Such a rare case of myoepithelioma in salivary glands present at an unusual location around the left orbit is presented here.

## Introduction

Myoepitheliomas of salivary glands are extremely rare, comprising approximately only 1–1.5% of all salivary gland tumors.,^2^ Myoepitheliomas were first described in 1943 as a variant of pleomorphic adenomas.^3^ In 1975, Stromeyer et al reported the first documented case of a malignant myoepithelioma. Two years later, Bauer called them adenomyoepitheliomas.^4^ Following the original description, numerous reports were published discussing whether myoepitheliomas originated de novo or as part of a mixed tumor. Such a rare case of myoepithelioma at an unusual location is presented here. These types of cases create a dilemma in the mind of the clinician.

## Case report

**Figure 1:**
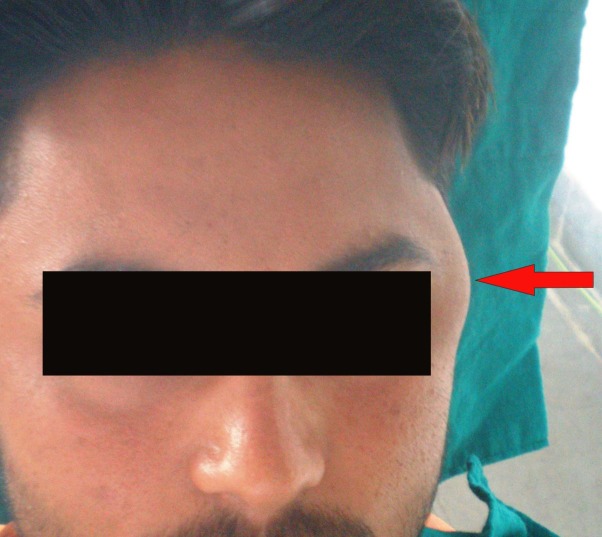
Extraoral examination revealing the swelling near the left orbit.

A 22-year-old male came with a complaint of a swelling on the left side of left eye since 1 year. The swelling was a small quiescent nodule approximately 1 year back and had gradually increased to attain the present size. There was no history of any trauma in this area with no history of any paresthesia or recent weight loss. Extra-oral examination revealed a well defined oval swelling approximately 4 cm in maximum diameter antero-posteriorly, present 1 cm lateral to outer canthus of left eye and ending approximately 6 cm in front of external auditory meatus, and measuring about 5 cm superoinferiorly. The overlying skin was smooth and normal in color. On palpation, the swelling was firm in consistency and was non-tender. It was not fixed to the underlying tissues or to the overlying skin. No lymph nodes were palpable (Figure 1). No radiographic or sonographic changes were noted.

**Figure 2:**
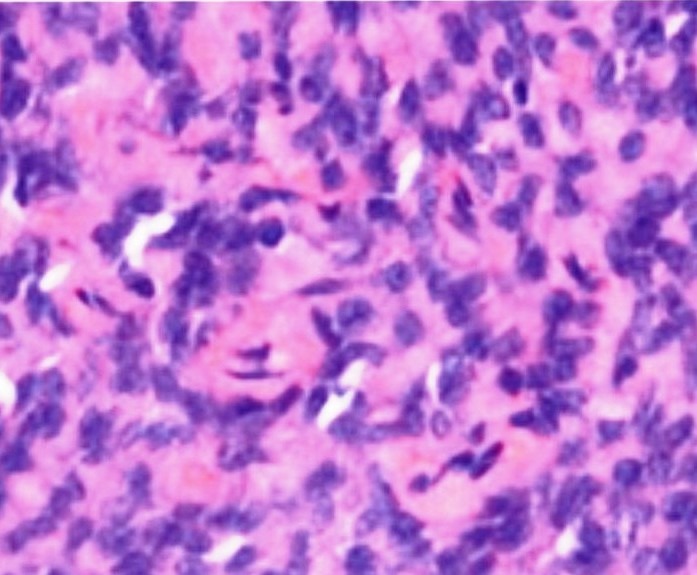
Histopathological image of the surgically excised lesion.

**Figure 3:**
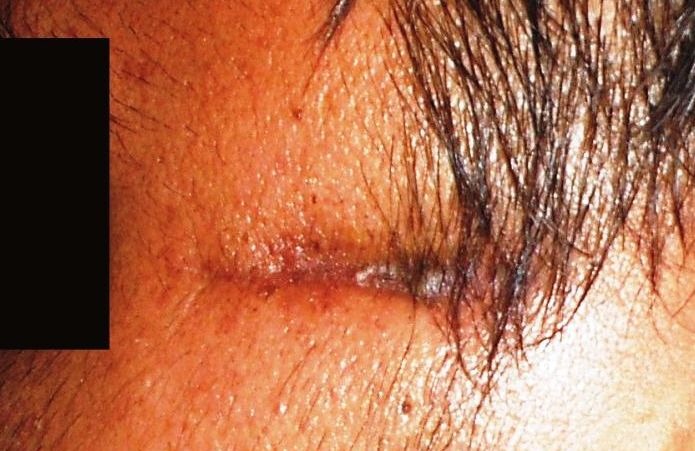
Healing of the surgical site post surgery.

The swelling was surgically excised and sent for histopathological examination, where it was diagnosed as myoepithelioma (Figure 2). The patient was followed up regularly (Figure 3). Five months after the surgery, the patient was well without evidence of recurrence or metastasis.

## Discussion

Myoepitheliomas account for less than 1.5% of all salivary gland tumors. A review of the literature through 1993 yielded approximately 100 cases. The number of case reports is increasing as pathologists have become more aware of its existence.^5^ The age and gender distribution of myoepithelioma is similar to that of mixed tumors; mostly occurring in 4^^th^^ to 6 ^th^ decade of life.^5^ The most common locations of myoepithelioma of the head and neck are the parotid gland (40%) and the palate (21%).^6^ Myoepitheliomas have been known to occur at various uncommon locations like maxillary sinus,^7^ and sinonasal cavity.^8^ Up until the present report, no similar case has been reported in the literature, making it a unique case owing to its uncommon location. There are no distinctive clinical features and, similar to most of the other salivary gland tumors, myoepitheliomas present as asymptomatic, slowly growing masses. Parotid lesions never cause facial nerve dysfunction or cervical lymphadenopathy, and those of the palate rarely ulcerate.

Microscopically, several growth patterns occur: solid, the most common, mixoid (pleomorphic adenoma like), reticular, and mixed. Cells can vary in histological appearance being spindle-shaped, plasmacytoid, and epitheloid; occasionally, epitheloid cells have clear cytoplasm. The different cellular compositions have no correlation to prognosis.^9 , 10^ The appearance of myoepithelioma on computed tomography (CT) imaging, as a well-defined enhancing mass, is not specific.^11^

The differential diagnoses of myoepitheliomas include pleomorphic adenoma and other mesenchymal neoplasms, nasal fibrosarcoma, fibromatosis of the sinonasal tract, neoplasms of smooth muscles, sinonasal myxoma, and embryonal rhabdomyosarcoma. Malignant myoepitheliomas have been described that infiltrate the surrounding tissues and even metastasize.^1^ Such myoepitheliomas can arise either de novo or develop ex-pleomorphic adenoma or ex-benign myoepitheliomas.^12 , 13^ These can be differentiated on the basis of radiographic or sonographic changes, which were absent in the present case. The treatment of a myoepithelioma is generally considered as complete surgical excision.^1^
